# Wearable Devices for Monitoring and Management of Comorbid Obstructive Sleep Apnea and Hypertension: Scoping Review

**DOI:** 10.2196/84506

**Published:** 2026-07-31

**Authors:** Hongyi Wang, Anran Cheng, Yusheng Li, Haining Zhang, Shun Fan, Rong Shen, Wei Zhang, Huanan Li, Jingui Wang

**Affiliations:** 1 Tuina Department, First Teaching Hospital of Tianjin University of Traditional Chinese Medicine, No. 88, Chang Ling Road, Li Qi Zhuang Jie, Xi Qing District, Tianjin, 300381, China, 86 22-27987000, 86 22-27986078; 2 National Clinical Research Center for Chinese Medicine, Tianjin, China; 3 Department of Sleep Medicine Center, Longhua Hospital Shanghai University of Traditional Chinese Medicine, Shanghai, China; 4 Anhui University of Traditional Chinese Medicine, Hefei, Anhui, China

**Keywords:** obstructive sleep apnea, hypertension, comorbidity, wearable devices, mHealth, continuous monitoring

## Abstract

**Background:**

As an established driver of hypertension, obstructive sleep apnea (OSA) generates a significant cardiovascular burden when both disorders are present. This intersection not only compromises nocturnal hemodynamics but also hampers long-term clinical management. Yet, current health care delivery rarely integrates the simultaneous and continuous tracking of these dual burdens. While wearable technology provides a noninvasive, pragmatic toolset for synchronized physiological monitoring, research remains largely siloed within single-disease frameworks. Consequently, clinical evidence supporting wearable applications specifically for comorbid populations remains sparse.

**Objective:**

In this scoping review, we summarized the current applications of wearable technology for comorbid OSA and hypertension. The analysis primarily outlines device categories, monitored physiological indicators, prevalent clinical scenarios, and existing challenges.

**Methods:**

The search for relevant literature spanned PubMed, Web of Science, Embase, and IEEE Xplore, covering the period from January 2015 to February 2026. Eligible studies included adults and used wearable or portable technologies to objectively track sleep- or respiratory-related indicators and cardiovascular/blood pressure metrics. Study selection, data charting, and evidence synthesis were conducted using a 2-reviewer process and descriptive and narrative approaches.

**Results:**

Our initial search yielded 739 records. Following title and abstract screening, we reviewed 54 full texts, ultimately finalizing a cohort of 13 eligible studies. Published between 2015 and 2026 across 9 countries, these articles capture data from 5596 participants. Most were observational studies and device validation studies. Evaluated device types included wrist-worn devices, fingertip contact devices, patch and single-lead devices, and multiparameter portable monitoring systems. The clinical applications mainly focused on screening and risk stratification for comorbid OSA and hypertension, monitoring of abnormal nocturnal blood pressure and hemodynamic changes, and cardiovascular risk assessment and remote longitudinal management. However, research remains sparse, and different devices vary greatly in reference standards, diagnostic thresholds, and validation pathways; therefore, their clinical translational value still requires further validation.

**Conclusions:**

Wearable devices may complement traditional assessment by providing continuous nocturnal and longitudinal data. However, at present, they are more suitable as auxiliary monitoring and risk warning tools in comorbidity management and cannot yet replace standard sleep studies or standard blood pressure monitoring. Future research should further shift from feasibility validation to large-sample, prospective, multicenter clinical studies in comorbid populations.

## Introduction

### Background

Obstructive sleep apnea (OSA) is one of the most common chronic respiratory diseases, affecting approximately 1 billion people worldwide, with the greatest management burden in China, the United States, Brazil, and India [1,2]. OSA has long been regarded as a disease that causes daytime sleepiness and reduced sleep quality. However, increasing evidence indicates that OSA is an independent risk factor for hypertension and a common cause of secondary hypertension [3]. The risk of incident hypertension is doubled in the population with OSA relative to healthy controls. OSA is also highly prevalent among patients with hypertension, particularly in more difficult-to-control cases [4,5]. These 2 conditions often coexist insidiously at night, and each apneic event is accompanied by marked hemodynamic fluctuations. This comorbid state is associated with a greater cardiovascular burden, abnormal nocturnal hemodynamics, and more complex long-term management, and it may also increase the lifetime risk of cardiovascular diseases such as coronary heart disease and stroke [6,7].

**Figure 1.**
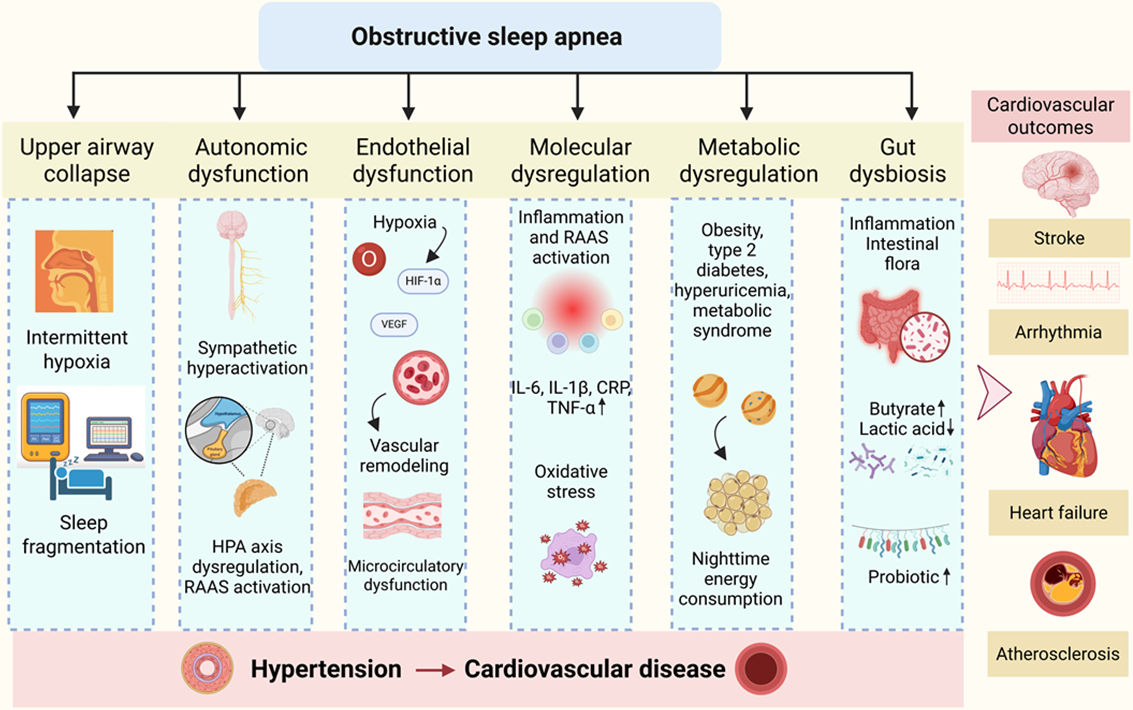
Proposed mechanisms linking obstructive sleep apnea and hypertension. CRP: C-reactive protein; HIF-1α: hypoxia-inducible factor 1 alpha; HPA: hypothalamic-pituitary-adrenal; IL-1β: interleukin 1 beta; IL-6: interleukin 6; RAAS: renin-angiotensin-aldosterone system; TNF-α: tumor necrosis factor alpha; VEGF: vascular endothelial growth factor.

The link between OSA and hypertension may involve multiple interrelated mechanisms (Figure 1). The most central mechanism is recurrent nocturnal hypoxia-reoxygenation and hypercapnia. These nocturnal abnormalities can continuously activate the sympathetic nervous system, promote peripheral vasoconstriction, and increase blood pressure [8]. Neuroendocrine dysregulation may also be involved, as abnormal hypothalamic-pituitary-adrenal axis rhythms can lead to elevated cortisol levels and further increase blood pressure [9]. Vascular impairment is further characterized by endothelial dysfunction, which serves as a mechanistic link between the two disorders. Intermittent hypoxic events induce the expression of hypoxia-inducible factors, subsequently modulating downstream mediators such as vascular endothelial growth factor. These molecular events promote structural remodeling and compromise endothelial integrity, leading to an elevation in peripheral resistance [10]. Vascular alterations are exacerbated by inflammatory and oxidative stress pathways. Sleep fragmentation and intermittent hypoxia facilitate a systemic inflammatory response, which is characterized by elevated concentrations of interleukin-6, tumor necrosis factor-α, and C-reactive protein. These inflammatory mediators contribute to the progression of underlying vascular dysfunction [11,12]. Shifts in gut microbiota and metabolic processes can compromise intestinal epithelial integrity and elevate vascular tone [13]. Metabolic factors, including obesity, dyslipidemia, and insulin resistance, further contribute to cardiovascular impairment and reinforce the bidirectional relationship between OSA and hypertension [14,15]. Since these pathophysiological processes are predominantly nocturnal and dynamic, single-point outpatient measurements or assessments focused on a single disease often fail to represent the complete clinical profile of this comorbidity.

At present, comorbid OSA and hypertension are still mostly managed separately across clinical specialties, with limited coordinated monitoring. Traditional data collection relies heavily on patient self-report or occasional in-hospital examinations and cannot provide real-time monitoring [16]. The lack of key continuous nocturnal data makes it difficult to capture the dynamic relationship between OSA and hypertension. In response to these clinical needs, wearable devices have provided new technologies for the continuous monitoring and management of comorbid OSA and hypertension. These devices usually integrate miniaturized sensors into watches, wristbands, skin patches, rings, and glasses, and can continuously and noninvasively collect multidimensional physiological signals such as electrocardiography, heart rate, heart rate variability (HRV), blood pressure, peripheral oxygen saturation, and sleep [16]. Wearable devices are designed for long-term wear and can measure changes in indicators during both daytime and sleep, thereby filling the gap in continuous nocturnal data acquisition in traditional assessment and better reflecting the dynamic relationship between OSA-related events and blood pressure fluctuations [17,18]. Wearable devices are expected to provide more timely and objective information for the screening, monitoring, and long-term management of this comorbid condition.

In recent years, reviews on the application of wearable devices in sleep monitoring, blood pressure monitoring, and chronic disease management have continued to increase. Most existing reviews have focused on a single-disease context of OSA or hypertension, or have only addressed devices, algorithms, and sensing signals [19-22]. There remains a lack of reviews that integrate devices, indicators, validation pathways, and clinical applications within the context of comorbidity management, which limits the overall understanding of their value, applicability, and current limitations among researchers and clinicians.

### Aims

This gap in the literature has hindered researchers’ understanding of the technology. Therefore, we conducted a scoping review to evaluate the current applications of wearable devices in the screening and management of comorbid OSA and hypertension, analyze recent developments and existing challenges, and inform future research directions.

We specifically addressed four aims: (1) to categorize wearable technologies used for the screening and management of concurrent OSA and hypertension, (2) to delineate the physiological parameters these tools monitor to evaluate respiratory and cardiovascular metrics, (3) to describe the clinical settings for device implementation and their reported practical use, and (4) to evaluate prevailing technical and methodological barriers, specifically regarding reference standards and clinical translation.

## Methods

### Overview

A scoping review approach was adopted in this study to address the substantial heterogeneity in device types, monitored indicators, and clinical application scenarios across the research questions, as well as the evidence gaps in previous reviews. The Arksey and O’Malley framework and the Preferred Reporting Items for Systematic Reviews and Meta-Analyses extension for Scoping Reviews (PRISMA-ScR) guidelines guided the methodological design and reporting of this scoping review. Related reporting checklists and structural details are available in Checklist 1 [23].

### Inclusion and Exclusion Criteria

The inclusion criteria were as follows: (1) clinical studies involving adults (aged ≥18 years); (2) studies in which the included population was explicitly a comorbid OSA and hypertension population, or studies in which the population had a single disease but both sleep- or respiratory-related indicators and blood pressure/cardiovascular indicators were assessed to evaluate the relationship between the 2 conditions or their comorbidity-related clinical applications; (3) studies using noninvasive wearable or portable devices to obtain at least 1 type of objective physiological monitoring data; (4) original studies published in English; and (5) studies with accessible full text. The exclusion criteria were as follows: (1) reviews, systematic reviews, meta-analyses, study protocols, case reports, animal experiments, commentaries, conference abstracts, and articles without full text were excluded; (2) studies that focused only on OSA or only on hypertension without objectively assessing disease-related indicators of the other condition; (3) studies that used only traditional in-hospital or laboratory monitoring without wearable or portable monitoring data; and (4) studies that were not clinically or practically validated based on real human data were excluded.

### Data Sources and Search Strategy

We comprehensively searched 4 databases, including PubMed, Web of Science, Embase, and IEEE Xplore. Original studies on wearable devices in the management of comorbid OSA and hypertension published between January 1, 2015, and February 28, 2026, were searched, and the final database search was completed on February 28, 2026. The search strategy included MeSH and free-text terms related to wearable devices and monitoring technologies (eg, “wearable electronics,” “portable devices,” “smartwatches,” “actigraphs,” “photoplethysmography,” “remote monitoring,” and “continuous monitoring”), OSA (eg, “obstructive sleep apnea,” “sleep apnea,” “sleep-disordered breathing,” and “snoring”), and hypertension (eg, “hypertension” and “ambulatory blood pressure monitoring”); the full search strategy is provided in Multimedia Appendix 1. To ensure the comprehensiveness of the literature collection, we manually traced the reference lists of all finally included studies. All retrieved records were imported into EndNote 21 (Clarivate) for deduplication and screening, and duplicate records were manually checked.

### Study Selection

Two researchers (HW and AC) independently conducted the initial screening of titles and abstracts to exclude records that did not meet the criteria. Following the preliminary screen, the remaining records underwent a complete full-text appraisal to confirm alignment with the predefined eligibility framework. Selection-related discrepancies were settled through consensus-based deliberation involving a third investigator (YL).

### Data Extraction and Charting

Two authors (HW and AC) jointly developed a standardized data-extraction and charting form based on the aims of this review and the predefined research questions. Before full data extraction, the 2 authors piloted the form on 3 randomly selected included studies to calibrate the definitions of key variables and classification rules and to improve consistency between reviewers. Data charting was treated as an iterative process; during piloting and discussion, the categories and recording rules in the extraction form were refined and then applied consistently in the final form. Subsequently, both authors independently extracted and charted data from all included studies. Disagreements were first resolved through discussion and consensus between the 2 authors; if consensus could not be reached, a third researcher (YL) was consulted.

**Figure 2.**
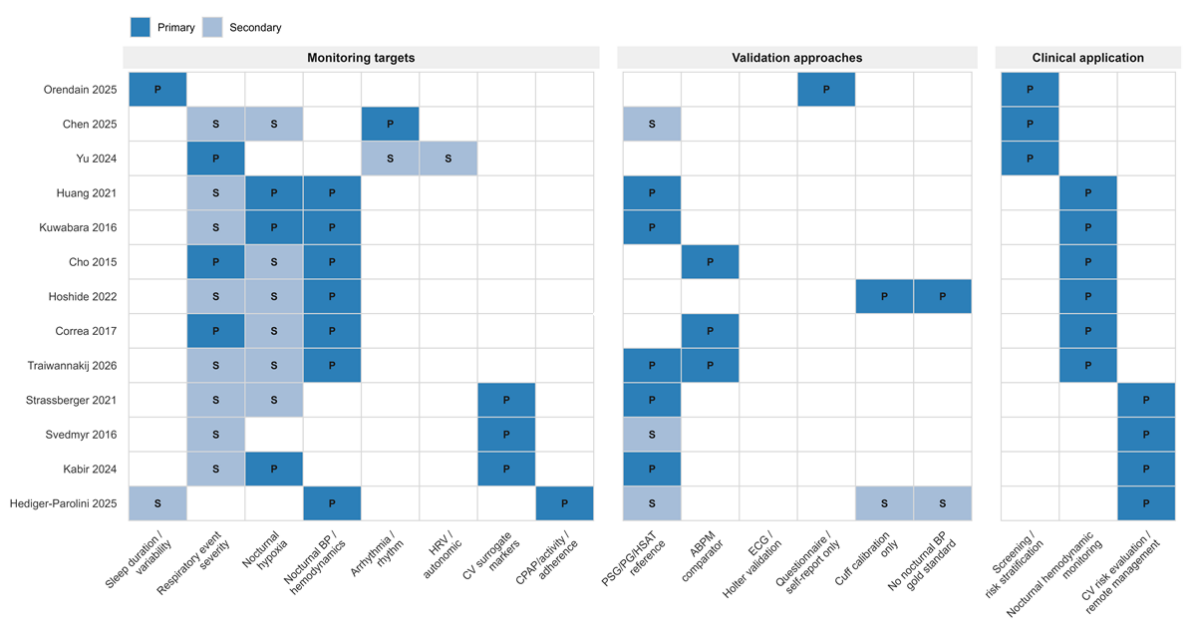
Study-by-dimension matrix of monitoring targets, validation approaches, and clinical applications across the included studies [24-36]. P indicates a primary focus, and S indicates a secondary or supportive feature. Blank cells indicate that the dimension was not central to the study. ABPM: ambulatory blood pressure monitoring; BP: blood pressure; CPAP: continuous positive airway pressure; CV: cardiovascular; ECG: electrocardiography; HRV: heart rate variability; HSAT: home sleep apnea test; PG: polygraphy; PSG: polysomnography.

We extracted study-level information across 5 domains: (1) study characteristics, including author, publication year, country, study design, sample size, participant characteristics, and study setting; (2) device characteristics, including wearable device type, sensing modality, and continuous monitoring capability; (3) physiological monitoring parameters, including sleep- or respiratory-related indicators and cardiovascular or hemodynamic indicators; (4) methodological characteristics, including reference standards, diagnostic or stratification thresholds, and validation design; and (5) clinical characteristics, including clinical application scenarios and study limitations. For studies involving multiple monitoring targets or application scenarios, the research team determined the primary focus according to the study objective, main outcome, and emphasis of reported findings, while also recording secondary or supportive features. The charted data were used to develop Table 1, Figure 2 [24-36], and Multimedia Appendices 2–4.

**Table 1.** Summary of study characteristics. Categories are not mutually exclusive; individual studies may contribute to multiple rows. For studies involving multiple monitoring targets or clinical applications, classification was based on the primary study objective, main outcome, and emphasis of reported findings. Respiratory event frequency metrics included AHI^a^, REI^b^, ODI^c^, or algorithm-estimated equivalents. Nocturnal BP^d^ variables included mean BP, peak BP, morning BP surge, BP variability, beat-to-beat BP, and cuffless BP estimates. Reference standards and assessment pathways were recorded as reported in each study.

Category	Studies, n (%)	References
Study design		
Observational studies	8 (62)	[24-31]
Device validation studies	4 (31)	[32-35]
Feasibility studies	1 (8)	[36]
Study setting/data source		
Sleep laboratory or hospital-based studies	9 (69)	[25-28,30,32-35]
Home or remote wearable monitoring studies	3 (23)	[24,29,36]
Community-based or real-world screening studies	3 (23)	[24,29,31]
Population/clinical context		
Suspected OSA^e^/screening populations	9 (69)	[24,27-29,32-36]
Confirmed OSA	3 (23)	[27,28,33]
CPAP-treated^f^ OSA	1 (8)	[36]
Primary hypertension	1 (8)	[25]
Older adults with hypertension	1 (8)	[31]
Obese adults at risk of abnormal nocturnal BP	1 (8)	[26]
Device type		
Wrist-worn devices	3 (23)	[24,29,36]
Fingertip contact devices	4 (31)	[25,26,30,34]
Patch and single-lead devices	2 (15)	[28,31]
Multiparameter portable monitoring systems	4 (31)	[27,32,33,35]
Sensor technologies		
PAT-based^g^ wearable studies	2 (15)	[25,26]
PPG/pulse^h^ oximetry–based wearable studies	8 (62)	[24,27,30,32-36]
Single-lead ECG–based^i^ screening studies	1 (8)	[31]
PTT-derived^j^ cuffless BP monitoring studies	2 (15)	[32,35]
Pulse wave/vascular analysis studies	2 (15)	[30,34]
Acoustic/tracheal sound wearable studies	1 (8)	[28]
Consumer sleep/activity tracker studies	1 (8)	[29]
Sleep and respiratory variables		
Sleep duration or sleep-variability metrics	3 (23)	[26,29,36]
Respiratory event frequency metrics (AHI/REI/ODI or equivalents)	12 (92)	[24-28,30-36]
Nocturnal hypoxia metrics (eg, SpO_2_^k^ nadir, T<90, ODT^l^)	9 (69)	[24-28,32-35]
Event- or stage-specific analyses	4 (31)	[27,28,33,35]
Cardiovascular variables		
Nocturnal BP level, surge, or variability	7 (54)	[25-27,32,33,35,36]
Beat-to-beat or continuous BP monitoring	3 (23)	[27,32,36]
Arrhythmia or rhythm abnormality monitoring	2 (15)	[24,31]
HRV^m^ or autonomic markers	2 (15)	[30,31]
Pulse wave or vascular stiffness markers	2 (15)	[30,34]
Reference standard/assessment pathway		
PSG^n^, polygraphy, or HSAT^o^	8 (62)	[24,27,28,32-36]
ABPM^p^	3 (23)	[25,26,35]
ECG/Holter	1 (8)	[31]
Questionnaire or self-report	1 (8)	[29]
Office cuff calibration without an external nocturnal BP gold standard	2 (15)	[32,36]
No external nocturnal BP gold standard despite BP-focused monitoring	3 (23)	[27,32,33]
Clinical application		
OSA comorbidity screening and risk stratification	3 (23)	[24,29,31]
Nocturnal BP abnormality and hemodynamic monitoring	6 (46)	[25-27,32,33,35]
CV^q^ risk evaluation and remote longitudinal management	4 (31)	[28,30,34,36]

a AHI: apnea-hypopnea index.

b REI: respiratory event index.

c ODI: oxygen desaturation index.

d BP: blood pressure.

e OSA: obstructive sleep apnea.

f CPAP: continuous positive airway pressure.

g PAT: peripheral arterial tonometry.

h PPG: photoplethysmography.

i ECG: electrocardiography.

j PTT: pulse transit time.

k SpO_2_: peripheral oxygen saturation.

l ODT: oxygen desaturation time.

m HRV: heart rate variability.

n PSG: polysomnography.

o HSAT: home sleep apnea test.

p ABPM: ambulatory blood pressure monitoring.

q CV: cardiovascular.

### Synthesis of Results

We synthesized the charted data using descriptive analysis and narrative synthesis. Using the charted data, we grouped similar findings into common themes. Information related to wearable device applications, sensing modalities, monitored indicators, reference standards, validation pathways, and clinical uses was described and organized. We did not prespecify the number of thematic categories or restrict the number of categories to which each study could contribute, in order to capture the diversity of the current evidence in terms of device types, monitoring targets, validation approaches, and clinical application scenarios. For each study, we reported the device characteristics, monitored variables, diagnostic or stratification thresholds, reference standards, and main findings as closely as possible to the original report, including information provided in supplementary materials where available. Similar content was then grouped into common themes without changing the meaning of the original studies.

Because the included studies were markedly heterogeneous in terms of wearable device types, sensing technologies, outcome assessment indicators, and measurement settings, we did not attempt to quantitatively pool the data or conduct a meta-analysis. Instead, the key study information, relevant findings, and methodological characteristics reported in each study were summarized and recorded. This approach was consistent with the purpose of a scoping review, which was to summarize study characteristics, identify evidence gaps, and clarify directions for future research rather than to estimate pooled effect sizes. Therefore, no formal risk-of-bias assessment or methodological quality appraisal was conducted for the included studies; relevant methodological and practical limitations were summarized in the Results and Discussion sections.

## Results

### Study Selection

**Figure 3.**
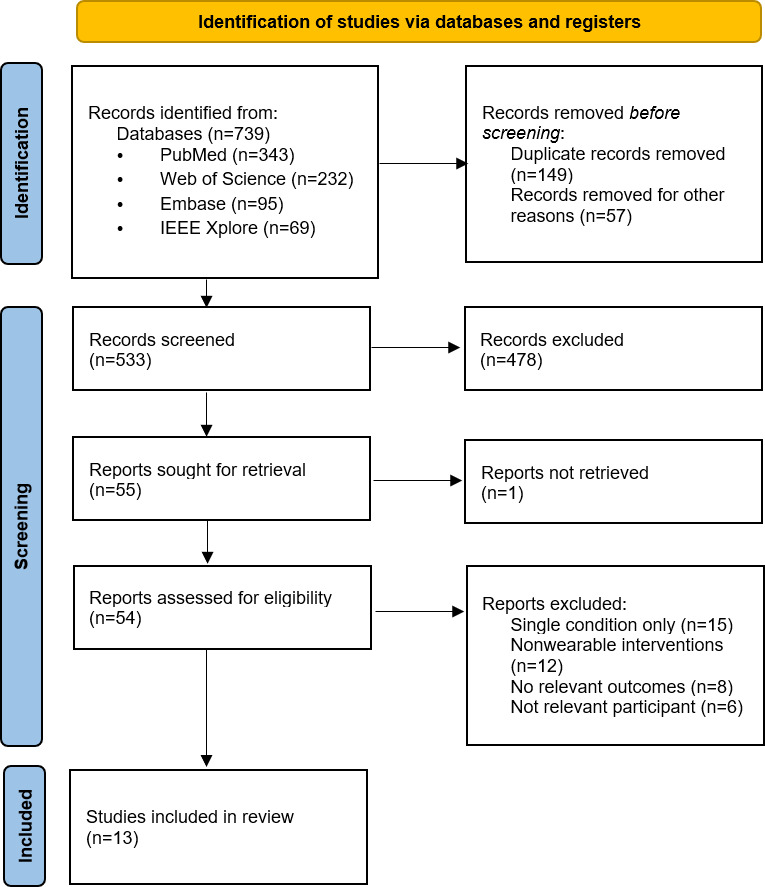
PRISMA (Preferred Reporting Items for Systematic Reviews and Meta-Analyses) flow diagram of study selection.

The PRISMA (Preferred Reporting Items for Systematic Reviews and Meta-Analyses) flow diagram (Figure 3) outlines the study selection process. A total of 739 studies were identified from the 4 databases. After removal of duplicates and records excluded for other reasons, 533 studies entered the title and abstract screening stage, and 478 articles were excluded after screening. Full texts of 55 articles were sought and assessed, of which 1 could not be retrieved. After full-text screening of the remaining 54 articles, 41 studies were excluded. Finally, 13 studies were considered eligible.

### Characteristics of Included Studies

The 13 studies included in this scoping review were published between 2015 and 2026 and were conducted in 9 countries or regions, namely China, Japan, South Korea, Thailand, the United States, Canada, Brazil, Switzerland, and Germany/Sweden, involving a total of 5596 participants. Among them, 1 real-world study included initial screening data from more than 1 million users [24]. The included literature comprised 8 (62%) observational studies [24-31], 4 (31%) device validation studies [32-35], and 1 (8%) feasibility study [36]. The study populations were markedly heterogeneous and covered multiple clinical scenarios in the management of comorbid OSA and hypertension. Participants included patients with OSA at different stages, ranging from suspected screening and confirmed diagnosis to treatment with continuous positive airway pressure (CPAP), as well as patients with primary hypertension, older adults with hypertension, and obese adults at risk of abnormal nocturnal blood pressure. A summary of the characteristics of the included studies is shown in Table 1, and detailed information on the included studies is provided in Multimedia Appendix 2.

### Wearable Device Types

The 13 included studies used various types of wearable devices, showing the characteristics of portability and continuous monitoring; detailed device information is provided in Multimedia Appendix 3. These included wrist-worn devices (n=3, 23%) [24,29,36], fingertip contact devices (n=4, 31%) [25,26,30,34], patch and single-lead devices (n=2, 15%) [28,31], and multiparameter portable monitoring systems (n=4, 31%) [27,32,33,35]. Wrist-worn devices mainly included smartwatches, wristbands, and activity trackers, and because of their greater convenience and practicality, they were mainly used for community screening, daily monitoring, and remote follow-up. Multiple studies used fingertip contact devices to collect nocturnal respiratory events and pulse wave signals in order to extract key characteristic parameters such as oxygen desaturation index (ODI), HRV, pulse propagation time (PPT), and pulse transit time (PTT). Patch and single-lead devices were used for the noninvasive continuous acquisition of acoustic or electrocardiographic signals. Some studies also applied multiparameter portable systems that combined sleep respiratory monitoring with continuous ambulatory blood pressure monitoring (ABPM) to synchronously assess nocturnal respiratory events and their related hemodynamic changes.

### Sensor Technologies

The included studies used multiple sensing technologies for the noninvasive acquisition of sleep and cardiovascular parameters. Optical sensing was the most commonly used technology, and photoplethysmography and pulse oximetry were the most widely applied, mainly for recording hypoxic events, hemodynamic fluctuations, and assessing nocturnal cardiovascular risk [24,27,30,34,36]. Two studies further combined optical and electrical signal sensing and extracted PTT by acquiring electrocardiography and fingertip photoplethysmography signals to achieve cuffless blood pressure estimation, thereby extending monitoring from OSA to the preliminary assessment of nocturnal hypertension or masked hypertension [32,35]. Some studies also used peripheral arterial tonometry (PAT), acoustic sensing, and actigraphy-related sensing technologies. Cho et al [25] and Correa et al [26] used PAT-related signals to assess changes in peripheral vascular tone and sympathetic nervous activity associated with sleep-related respiratory events [25,26]. Accelerometers were commonly used for activity state identification, sleep staging, and variability analysis [29,36]. Kabir et al [28] collected tracheal breath sounds and heart sound signals using miniature microphones and accelerometers and analyzed them synchronously with polysomnography.

### Monitored Parameters

Based on the multimodal sensing technologies described above, current evidence suggests that some wearable devices can simultaneously capture sleep-related respiratory parameters and hemodynamic parameters. In the sleep-respiratory domain, the devices were mainly used to assess OSA severity and nocturnal hypoxic burden. The monitored indicators included the apnea-hypopnea index (AHI) or respiratory event index (REI), ODI, minimum or mean peripheral oxygen saturation (SpO_2_), sleep time with oxygen saturation below 90%, or other indicators of hypoxic exposure [24-27,30-33,35]. Some studies obtained sleep rhythm indicators through long-term monitoring, including total sleep time, sleep efficiency, and sleep variability [26,29,30]. Other studies focused on respiratory signal features such as nasal airflow, snoring, and thoracoabdominal respiratory effort [28,32]. In comparison, cardiovascular and hemodynamic parameters may better reflect the comorbidity characteristics of OSA and hypertension. Wearable devices were able not only to derive conventional indicators such as resting heart rate and HRV [29,31] but also to obtain continuous beat-to-beat blood pressure, PTT-based nocturnal ambulatory blood pressure [27,32,35], systolic blood pressure peaks triggered by hypoxic events [33], PAT amplitude attenuation caused by sympathetic activation [25,26], and PPT, which is related to vascular stiffness or nocturnal cardiovascular burden [30]. In addition, some studies also recorded changes in heart sound intensity and nocturnal abnormal arrhythmic events [24,28,31]. Overall, there was marked heterogeneity across the included studies in terms of reference standards, assessment pathways, and diagnostic or stratification thresholds, including different assessment methods such as polysomnography, home sleep apnea testing (HSAT), ABPM, and questionnaire tools. Blood pressure thresholds are reported as used in the original studies for diagnostic or stratification purposes; treatment targets were not synthesized because most included studies focused on screening and monitoring rather than treatment-goal attainment. Therefore, the reported device performance should be interpreted objectively in the context of specific study settings (Multimedia Appendix 4).

### Clinical Applications

#### Overview

Evidence suggests that wearable technology for comorbid OSA and hypertension management largely focuses on three functional areas: initial comorbidity screening and risk stratification in community and home settings, continuous monitoring of abnormal nocturnal hemodynamics and changes in respiratory events, and multimodal cardiovascular risk assessment and exploration of remote management. Figure 2 summarizes the monitoring targets, validation approaches, and major clinical application scenarios across the included studies.

#### Screening and Risk Stratification for Comorbid OSA and Hypertension

Three studies involved the application of wearable devices in the screening and risk stratification of comorbid OSA and hypertension [24,29,31]. Existing evidence suggests that wearable devices have been increasingly applied to the early identification and risk stratification of this comorbidity. In a large real-world screening study, wrist-worn smart devices were used for initial screening in 1,056,494 users, identifying 19,563 individuals at high risk for OSA, and 1054 patients with OSA were confirmed among those who subsequently underwent HSAT or polysomnography evaluation; the devices could also simultaneously indicate the risk of arrhythmia [24]. A study including 2573 older adults with hypertension in primary care settings found that continuous monitoring based on wearable electrocardiography and the autocorrelated wave detection with adaptive threshold (ACAT) algorithm could be used for community-based initial screening of OSA and could help improve the identification of individuals at high risk for moderate to severe OSA [31]. Another study analyzed long-term sleep data collected by wearable devices together with hypertension information and found that increased sleep variability was associated with higher OSA risk and increased hypertension risk, suggesting that wearable devices may be used for the preliminary identification of comorbidity risk [29]. The application of wearable devices is no longer limited to the detection of OSA alone but has extended to the preliminary identification of comorbidity risk. However, their role still lies in early identification and referral stratification, and they cannot replace standard sleep monitoring or the diagnostic pathway for OSA.

#### Monitoring of Abnormal Nocturnal Blood Pressure and Hemodynamic Changes

A total of 6 studies involved the monitoring of abnormal nocturnal blood pressure and respiratory event–related hemodynamic changes [25-27,32,33,35]. Wearable devices can not only monitor nocturnal blood pressure but also record the dynamic relationship between OSA-related hypoxic events and abnormal blood pressure fluctuations. Multiple studies found that the more severe the hypoxic events, the more pronounced the increase in nocturnal systolic blood pressure and blood pressure variability, suggesting that OSA-related hemodynamic changes are often event-triggered [27,33]. In a multicenter study of 330 patients with suspected sleep-disordered breathing, beat-to-beat PTT-estimated blood pressure was compared with fixed-interval PTT-estimated blood pressure. The results showed that the 2 methods were generally similar in mean blood pressure and blood pressure variability measures, but beat-to-beat monitoring was more sensitive in capturing maximum and minimum values [32]. Another study, using 24-hour ABPM as the reference, found that the PTT estimation method based on combined electrocardiography and photoplethysmography signals was only weakly correlated with nocturnal ABPM and tended to overestimate nocturnal blood pressure in patients with moderate to severe OSA, indicating that a gap in agreement with the gold-standard ABPM still remains [35].

#### Cardiovascular Risk Assessment and Remote Longitudinal Management

A total of 4 studies involved wearable devices in cardiovascular risk assessment and remote longitudinal management in this comorbidity [28,30,34,36]. In the assessment of hypertension and cardiovascular risk, 2 studies involving populations with suspected OSA showed that wearable devices could provide warning indicators such as the cardiac risk index, pulse wave attenuation index, and PPT. The studies reported that the cardiac risk index may be superior to conventional OSA severity indicators in identifying individuals at high cardiovascular risk, and that the derivation of these parameters may help identify high-risk cardiovascular phenotypes of OSA associated with increased arterial stiffness [30,34]. Another study found that heart sound intensity increased significantly after the termination of respiratory events and was closely associated with the degree of hypoxia and the concomitant fluctuations in blood pressure and heart rate, suggesting that patch-based devices may complement the assessment of the comorbid cardiovascular burden related to OSA from the perspective of cardiac mechanical load [28]. In terms of remote longitudinal management, a feasibility study including only 10 patients with OSA receiving CPAP treatment found that home monitoring using a multimodal wearable system could be used for the continuous observation of sleep, activity, and blood pressure changes and may support the remote management of intervention adherence and blood pressure fluctuations [36].

### Technical Limitations and Challenges

Technical and translational obstacles persist in the application of wearables for managing comorbid OSA and hypertension. A total of 5 (38%) studies lacked adequate reference standard validation or used different assessment pathways, which limited the comparability of results across different devices and algorithms [25,29-32]. Selection biases attributable to voluntary enrollment, specific referral cohorts, and single-center designs were identified across multiple studies, restricting the generalizability of observations across diverse demographics and clinical outcomes. Methodological inconsistencies were observed in specific reports, such as the reliance on self-reported hypertension status (1/13, 8%) rather than clinical diagnostic verification [29]. Another study involved cardiovascular risk assessment derived from questionnaires and telephone-based follow-ups, a process susceptible to subjective bias compared to objective clinical measurements [24]. These observations support the use of wearables for risk stratification rather than as direct diagnostic substitutes. Data from Traiwannakij et al [35] indicated weak concordance between PTT-derived cuffless blood pressure monitoring and gold-standard ABPM; specifically, a systematic overestimation of nocturnal values was observed among patients with moderate to severe OSA. In addition, in remote or multimodal monitoring settings, obstacles such as reduced quality of nocturnal blood pressure data, difficulty in device calibration, and reliance on manual processing for cross-device data synchronization also remain, all of which limit the clinical application of wearable devices [36].

## Discussion

### Principal Findings

To our knowledge, this scoping review is the first review to systematically summarize the evidence on the application of wearable devices in the screening, monitoring, and management of OSA and hypertension from the perspective of this comorbidity. A total of 13 studies were included, involving 4 types of wearable devices, namely wrist-worn devices, fingertip contact devices, patch and single-lead devices, and multiparameter portable monitoring systems, covering a range of application scenarios from clinical validation to real home settings. Previous reviews have explored the feasibility of wearable devices in the monitoring of OSA or hypertension alone. From the perspective of comorbidity, this review further integrated the existing evidence on sleep-related respiratory signals and nocturnal hemodynamic information in the assessment of this comorbidity. The findings showed that wearable devices can simultaneously obtain sleep-related respiratory parameters and cardiovascular-related indicators, and have shown preliminary application value in the identification of populations at high risk for OSA, the preliminary identification of abnormal nocturnal blood pressure, cardiovascular risk stratification, and remote follow-up. However, the current evidence remains limited and heterogeneous, particularly in terms of reference standards, diagnostic thresholds, and validation pathways. The following sections further discuss the clinical significance, technical boundaries, and future directions of current sleep and blood pressure monitoring.

### Value of Sleep and Blood Pressure Monitoring From a Comorbidity Perspective

The clinical evaluation of OSA has long centered on AHI and the hypoxia and sleep architecture indicators obtained from conventional sleep monitoring. However, from the perspective of comorbid OSA and hypertension, sleep monitoring alone is insufficient to fully reflect the true clinical significance of OSA. AHI and REI mainly reflect the frequency of respiratory events and are less able to capture information more closely related to cardiovascular risk, such as nocturnal hypoxic burden, autonomic nervous responses, and abnormal circadian blood pressure patterns [6,8]. Comorbidity management does not simply mean adding more monitoring indicators, but rather incorporating the nocturnal cardiovascular burden underlying respiratory events into the overall assessment. Therefore, the focus of monitoring in comorbidity management should not be limited to AHI and ODI, but should also include indicators that better reflect the characteristics of OSA-related hypertension, such as hypoxic burden, respiratory effort burden, elevated nocturnal blood pressure, reduced nocturnal blood pressure dipping, abnormal morning blood pressure surge, and blood pressure variability. Traditional assessment mainly relies on patient recall or single in-hospital examinations and, in particular, lacks nocturnal data, making it difficult to effectively capture the dynamic relationship between OSA and hypertension [16]. The 24-hour ambulatory blood pressure and nocturnal blood pressure are closely associated with cardiovascular risk, and home morning blood pressure is closely associated with prognosis in patients with cardiovascular disease [37]. Traditional assessment may miss high-risk phenotypes such as masked hypertension, nondipper/riser blood pressure patterns, and morning blood pressure surge [5,38]. Reliance on a single sleep monitoring and routine outpatient assessment is therefore insufficient to fully identify the true risk of OSA-related hypertension. At the same time, OSA-related nocturnal hemodynamic changes may also manifest as autonomic dysfunction and increased blood pressure variability, suggesting that comorbidity management should further incorporate these related indicators [39]. Taken together, the assessment of comorbid OSA and hypertension should not remain limited to single examinations, but should further shift toward long-term dynamic monitoring.

The most direct clinical value of wearable devices lies in supporting long-term, longitudinal, and individualized monitoring in the management of comorbid OSA and hypertension. ABPM can monitor day-night blood pressure variability and detect masked hypertension, but it cannot assess daily and long-term blood pressure variability, including night-to-night variability; home blood pressure monitoring (HBPM) is suitable for tracking daily and long-term blood pressure trends, but measurements are mostly concentrated at fixed time points, such as in the morning and at night [40]. Single-night polysomnography is difficult to use to reflect OSA severity and its night-to-night variability, whereas ABPM and HBPM can provide blood pressure information, each still has limitations in long-term continuous dynamic tracking. Existing studies have shown through multinight sleep monitoring and repeated blood pressure measurements that the greater the night-to-night variability of OSA, the higher the risk of uncontrolled hypertension, and this association is independent of mean AHI [41]. Continuous monitoring may help more accurately identify hypertension risk and nocturnal vascular burden. For patients with OSA receiving CPAP treatment, wearable devices may support long-term adherence tracking and assist in evaluating posttreatment blood pressure improvement and cardiovascular benefit [36]. Consistent CPAP use correlates with lower rates of recurrent cardiovascular episodes, serving as an integral element of secondary prevention for the population with OSA [42]. For individuals presenting with both resistant hypertension and OSA, CPAP therapy facilitates reductions in 24-hour mean and nocturnal blood pressure and is more likely to correct nondipper/riser blood pressure patterns [43]. Therefore, the future value of wearable devices lies not only in improving the convenience of screening but also in providing continuous data support for treatment adherence tracking, blood pressure response assessment, and long-term cardiovascular risk stratification.

### Clinical Limitations and Technical Boundaries of Wearable Monitoring

At present, the value of wearable technologies is reflected more in screening, dynamic monitoring, and long-term follow-up rather than in replacing standard diagnosis, and polysomnography remains the gold standard for OSA. The included studies found that wearable devices are feasible for identifying high-risk populations in large-scale real-world studies and primary care screening studies and are more suitable for initial screening and risk stratification [24,31]. Consistent with previous studies, most wearable devices lack complete electroencephalography channels, making full sleep staging difficult and limiting the accurate identification of microarousal-related hypopnea events, respiratory effort, and more complex respiratory events [44,45]. Signal fidelity frequently degrades due to postural shifts, ambient noise, and motion artifacts [28]. Proprietary algorithms differ across manufacturers, complicating their generalization to patients with complex comorbidities [31,45].

Continuous home-based blood pressure tracking via cuffless systems does not currently serve as a full replacement for standard ABPM or HBPM protocols [35]. Persistent technical limitations such as signal instability and mandatory calibration requirements, alongside sensitivity to postural changes and localized skin perfusion variations, affect the precision of these measurements [46,47]. The primary technical challenge involves the consistent recording of 24-hour blood pressure fluctuations rather than the accuracy of isolated measurement approximations [48]. Although certain cuffless tools demonstrate acceptable performance during the day, their nocturnal estimates frequently lack precision and may fail to document transient pressure surges or circadian irregularities with sufficient reliability [27,48]. Inconsistencies in validation frameworks and algorithmic logic among manufacturers prevent data uniformity. Large-scale clinical datasets focused on blood pressure fluctuations specific to nocturnal OSA events also remain scarce. Therefore, cuffless blood pressure monitoring is currently better positioned as a tool for trend observation and risk warning than as a substitute for ABPM or HBPM.

### Multimodal Sensing and Missing-Modality Handling

Recent studies have shown that intelligent OSA identification technologies are gradually shifting from single-signal analysis to multimodal sensing fusion. By integrating visual signals, snoring audio, body movement information, and multiple electrophysiological signals, related models have shown greater accuracy than single-modality approaches in OSA identification and stratification [49]. To address sensor detachment, motion artifacts, and modality missingness during long-term wearable monitoring, some studies have also begun to explore missing-modality reconstruction strategies to improve model robustness under incomplete input conditions. For example, the MIMAR-OSA model was able to maintain relatively high monitoring performance under partial signal loss through multimodal data integration and missing-modality reconstruction [50]. However, these advances are currently still limited to the automatic identification, severity stratification, and sleep staging of OSA itself, and are mainly focused on improvements in signal processing, feature extraction, and algorithm robustness. They have not yet been extended to the identification of abnormal nocturnal blood pressure or the long-term management of comorbid OSA and hypertension. Therefore, such studies may be more suitable as supporting technologies for future digital management of this comorbidity, but prospective clinical validation in the setting of comorbid OSA and hypertension is still lacking.

### Exploration of a Sleep and Blood Pressure Closed-Loop Management Pathway

Existing studies have mainly involved digital care, wearable device monitoring, risk alert platforms, and long-term management, and a mature “sleep and blood pressure” closed-loop management model has not yet been established for comorbid OSA and hypertension [51,52]. Based on the included evidence, such a framework should at least involve 4 key components. At the input level, key information reflecting the comorbid state should be continuously acquired, including multinight sleep-related respiratory events, hypoxic burden, nocturnal blood pressure or blood pressure rhythm changes, HRV, and treatment adherence. At the output level, a more reasonable goal is not to directly generate an automated diagnosis, but to identify risk signals relevant to comorbidity management, such as suspected OSA-related nocturnal hypertension, persistent abnormal hypoxic burden, reduced CPAP adherence, or disordered nocturnal blood pressure rhythms. These risk alerts should primarily prompt clinicians to arrange polysomnography, supplement ABPM, or intensify remote follow-up, rather than delegating diagnostic or therapeutic decisions to autonomous algorithms. The safety boundaries of sleep and blood pressure management need to be clearly defined. Data outputs from wearable platforms operate as auxiliary indicators that do not replace gold-standard monitoring or bypass professional clinical appraisal. The use of wearables alongside CPAP remote monitoring is a feasible approach for home observation [36]. Future sleep and blood pressure closed-loop pathways are envisioned as auxiliary frameworks rather than fully autonomous diagnostic systems.

### Barriers to Clinical Implementation

Practical obstacles persist in the clinical application of wearables for patients with concurrent OSA and hypertension. The existence of continuous data streams does not automatically translate into improved management efficiency. Clinical staff still have limited training in devices and related algorithms, and the interpretation of device data and verification of abnormal alerts may create an additional workload. In addition, there is still a lack of standardized clinical pathways, evaluation frameworks, and reimbursement systems applicable to the management of comorbid OSA and hypertension, making these technologies difficult to implement in routine clinical practice. Moreover, patient adherence, motion artifacts, and reduced nocturnal signal quality may further affect their application in clinical settings.

Therefore, in future clinical translation, it may be necessary to gradually establish a minimum necessary dataset for the management of comorbid OSA and hypertension, including core physiological indicators, device algorithm metadata, signal quality, and adherence information, in order to reduce the problem that outputs from different platforms are difficult to compare or cannot be incorporated into the clinical interpretation workflow [53,54]. On this basis, core indicators and quality control standards should also be standardized to improve the transferability and traceability of data across different systems. Responsibility for data quality should be further clarified. For example, synchronization, quality labeling, and abnormal alert generation could be completed at the device or platform level, while the clinical team would review the alert results, thereby reducing the data overload and additional interpretation burden caused by continuous monitoring [52,55].

### Methodological Limitations

This scoping review has several methodological limitations. Only English-language studies were included, and gray literature and preprints were not included, which may have introduced language bias and publication bias. Second, because the current evidence on comorbid OSA and hypertension remains limited, this review included some studies conducted in single-disease populations that simultaneously assessed sleep- or respiratory-related indicators and blood pressure/cardiovascular indicators. Although this strategy expanded the scope of evidence covered, it also increased the heterogeneity across study populations, application scenarios, and outcome indicators. In addition, the number of included studies was limited, and some studies had small sample sizes, which restricted the robustness of the evidence. There was substantial heterogeneity across the included studies in terms of device types, reference standards, assessment pathways, and outcome definitions, and no meta-analysis was performed in this review. Overall, the conclusions of this review are more suitable for informing subsequent validation studies and real-world research, and they should still be interpreted with caution in specific clinical implementations.

### Future Directions

Based on the evidence gaps identified in this scoping review, future research should further shift from feasibility validation to clinically translational research in comorbidity management. Existing evidence mainly consists of observational studies and device validation studies and has focused primarily on whether devices can identify the risk of OSA and hypertension or record nocturnal physiological changes, with insufficient attention to the populations, indicators, and outcomes that are truly critical in comorbidity management. Future studies should more strongly emphasize large-sample, prospective, and multicenter research in clearly defined comorbid populations and should validate key nocturnal and long-term outcome indicators against reference standards such as polysomnography and ABPM. At the same time, the research focus should move beyond consistency with standard testing toward the effects of wearable devices on clinical outcomes such as blood pressure control, CPAP adherence, monitoring of nocturnal blood pressure patterns, and patient experience. Recent studies have also suggested that bedtime and circadian rhythm characteristics may be important additional factors for future comorbidity risk assessment [56]. Future comorbidity management should not be limited to the monitoring of apneic events alone, but should also incorporate sleep behavior, circadian rhythm characteristics, and hemodynamic changes into a comprehensive assessment. Finally, it will also be necessary to gradually establish core indicators, validation pathways, and reporting standards applicable to the management of comorbid OSA and hypertension, so that sleep and blood pressure data collected by wearable devices can better serve clinical care and be progressively integrated into standardized management pathways.

### Conclusions

Wearable devices support the screening, nocturnal monitoring, and remote follow-up of comorbid OSA and hypertension. Specifically, they provide the continuous nocturnal data and longitudinal follow-up metrics that are often absent in conventional diagnostic workflows. Their clinical value is gradually extending from the identification of single sleep events to the continuous observation of comorbidity-related nocturnal cardiovascular burden. However, the existing evidence still mainly comes from observational studies and device validation studies, and there is marked heterogeneity across devices in terms of reference standards, diagnostic thresholds, and validation pathways. At the current stage, wearable devices are more suitable as auxiliary monitoring and risk warning tools in comorbidity management and cannot replace standard sleep testing or standard blood pressure monitoring.

## Supplementary material

10.2196/84506 Multimedia Appendix 1 Full search strategies for all databases.

10.2196/84506 Multimedia Appendix 2 Characteristics of included studies.

10.2196/84506 Multimedia Appendix 3 Device characteristics and key findings.

10.2196/84506 Multimedia Appendix 4 Diagnostic or stratification thresholds.

10.2196/84506 Checklist 1 PRISMA-ScR checklist.

## Abbreviations

ABPM: ambulatory blood pressure monitoring
AHI: apnea-hypopnea index
CPAP: continuous positive airway pressure
HBPM: home blood pressure monitoring
HRV: heart rate variability
HSAT: home sleep apnea test
ODI: oxygen desaturation index
OSA: obstructive sleep apnea
PAT: peripheral arterial tonometry
PPT: pulse propagation time
PRISMA: Preferred Reporting Items for Systematic Reviews and Meta-Analyses
PRISMA-ScR: Preferred Reporting Items for Systematic Reviews and Meta-Analyses extension for Scoping Reviews
PTT: pulse transit time
REI: respiratory event index
SpO_2_: peripheral oxygen saturation
