# Supplementary material for: Wearable Devices for Monitoring and Management of Comorbid Obstructive Sleep Apnea and Hypertension: Scoping Review
Source: JMIR Mhealth Uhealth. 2026 Jul 31;14:e84506. doi: 10.2196/84506 (PMC13427068; doi:10.2196/84506)
Supplement: Multimedia Appendix 1 [file mhealth-v14-e84506-s001.docx]

## **Multimedia Appendix 1. Full search strategies for databases**

**Search executed on February 28, 2026.**

Searches were limited to English-language records published between January 1, 2015 and February 28, 2026.

**PubMed (n=343)**

(("Wearable Electronic Devices"[MeSH] OR "Telemedicine"[MeSH] OR "Monitoring, Ambulatory"[MeSH] OR "Actigraphy"[MeSH] OR "Photoplethysmography"[MeSH] OR wearable*[tw] OR smartwatch*[tw] OR "smart watch*"[tw] OR "smart ring*"[tw] OR cuffless[tw] OR biosensor*[tw] OR "continuous monitoring"[tw] OR "remote monitoring"[tw] OR mHealth[tw] OR eHealth[tw] OR "mobile health"[tw] OR actigraph*[tw] OR PPG[tw] OR "portable device*"[tw] OR "portable monitoring"[tw])) AND (("Sleep Apnea, Obstructive"[MeSH] OR "Sleep Apnea Syndromes"[MeSH] OR "obstructive sleep apnea"[tw] OR "obstructive sleep apnoea"[tw] OR OSAHS[tw] OR OSAS[tw] OR OSA[tw] OR "sleep-disordered breathing"[tw] OR SDB[tw] OR "nocturnal hypoxemia"[tw] OR apnea[tw] OR apnoea[tw] OR snoring[tw])) AND(("Hypertension"[MeSH] OR "Blood Pressure"[MeSH] OR "Blood Pressure Monitoring, Ambulatory"[MeSH] OR hypertension[tw] OR hypertensive[tw] OR "blood pressure"[tw] OR BP[tw]))

**Web of Science (n=232)**

TS=(("Wearable Electronic Devices" OR Telemedicine OR "Monitoring, Ambulatory" OR Actigraphy OR Photoplethysmography OR wearable* OR smartwatch* OR "smart watch*" OR "smart ring*" OR cuffless OR biosensor* OR "continuous monitoring" OR "remote monitoring" OR mHealth OR eHealth OR "mobile health" OR actigraph* OR PPG OR "portable device*" OR "portable monitoring")) AND TS=(("Sleep Apnea, Obstructive" OR "Sleep Apnea Syndromes" OR "obstructive sleep apnea" OR "obstructive sleep apnoea" OR OSAHS OR OSAS OR OSA OR "sleep-disordered breathing" OR SDB OR "nocturnal hypoxemia" OR apnea OR apnoea OR snoring)) AND TS=(("Hypertension" OR "Blood Pressure" OR "Blood Pressure Monitoring, Ambulatory" OR hypertension OR hypertensive OR "blood pressure" OR BP))

**IEEE Xplore(n=69)**

("All Metadata":wearable* OR "All Metadata":smartwatch* OR "All Metadata":"smart watch" OR "All Metadata":"smart ring" OR "All Metadata":cuffless OR "All Metadata":biosensor* OR "All Metadata":"continuous monitoring" OR "All Metadata":"remote monitoring" OR "All Metadata":mHealth OR "All Metadata":PPG) AND ("All Metadata":"sleep apnea" OR "All Metadata":OSAHS OR "All Metadata":OSAS OR "All Metadata":OSA OR "All Metadata":"sleep-disordered breathing" OR "All Metadata":apnea OR "All Metadata":snoring) AND ("All Metadata":hypertension OR "All Metadata":"blood pressure")

**Embase (n=95)**

(('obstructive sleep apnea' OR 'obstructive sleep apnoea' OR osahs OR osas OR osa OR 'sleep-disordered breathing') NEAR/10 (hypertension OR hypertensive OR 'blood pressure')) AND (wearable* OR smartwatch* OR 'smart watch*' OR 'smart ring*' OR cuffless OR actigraph* OR photoplethysmograph* OR 'wearable electronic device*')
