# Supplementary material for: Wearable Devices for Monitoring and Management of Comorbid Obstructive Sleep Apnea and Hypertension: Scoping Review
Source: JMIR Mhealth Uhealth. 2026 Jul 31;14:e84506. doi: 10.2196/84506 (PMC13427068; doi:10.2196/84506)
Supplement: Multimedia Appendix 2 [file mhealth-v14-e84506-s002.docx]

Baseline characteristics of the included studies.

| **Author, year** | **Country** | **Study design** | **Sample size** | **Age** | **Sex(M/F, n)** | **Population characteristics** | **OSA status** | **Hypertension status** | **Setting/data source** | **Monitoring duration** |
| --- | --- | --- | --- | --- | --- | --- | --- | --- | --- | --- |
| Huang et al., 2021 | China | Prospective single-center pilot study | 30 | 54.9 ± 11.7 | 24/6 | Adults with confirmed OSA and concurrent continuous BP assessment | Confirmed OSA (REI >= 5): mild 14, moderate 6, severe 10 | Hypertension excluded | Single-center overnight monitoring using the Morpheus Ox portable sleep monitor and CNAP beat-to-beat BP monitor | 1 night |
| Strassberger et al., 2021 | Germany/Sweden | Multicenter observational validation study | 358 | 55 ± 13 | male 64% | Patients with suspected OSA and concurrent cardiovascular risk assessment | Suspected OSA; median AHI 13 [5-26] events/h | Not separately reported; included in CV risk profiling | Five tertiary sleep centers; finger pulse oximeter module (ChipOx) used alongside standard overnight PG/PSG | 1 night |
| Traiwannakij et al., 2026 | Thailand | Prospective clinical study | 66 | 47, median(IQR 36 - 60) | 26/40 | Adults with suspected OSA undergoing full-night PSG and next-day ABPM | 63/66 had OSA: mild 19, moderate 25, severe 19 | Prior hypertension 27/66 (40.9%) | Single-center sleep laboratory; full-night PSG followed by SOMNOtouch NIBP cuffless monitoring and next-day WatchBP 03 ABPM | 1 night + 24 h |
| Orendain et al., 2025 | United States | Decentralized prospective cohort study | 391 | 48.1 ± 13.6 | 117/247; 27 missing | Community-dwelling adults with Fitbit-derived longitudinal sleep data | Berlin high-risk OSA 179/372 (48.1%) | Self-reported hypertension 92/346 (26.6%) | Nationwide smartphone-based REFRESH study with Fitbit-linked data | >=14 days to 4 years (mean about 2 years) |
| Kuwabara et al., 2016 | Japan | Prospective observational reproducibility study | 147 | 59.4 ± 13.7 | 127/20 | Adults with suspected OSA undergoing PSG and triggered nocturnal BP monitoring | Screening ODI > 15/h plus symptoms; mild 9.5%, moderate 29.9%, severe 60.6% | Hypertension in 62.6% | Sleep laboratory; PSG with triggered nocturnal BP monitoring | 2 consecutive nights |
| Cho et al., 2015 | South Korea | Prospective cross-sectional study | 58 (OSA group: 35; non-OSA group: 23) | 51.7 ± 11.3 | 32/26 | Never-treated essential hypertension patients with simultaneous portable sleep and ambulatory BP monitoring | OSA in 35/58; AHI >= 5 by Watch-PAT 200 | 100% never-treated essential hypertension | Single-center simultaneous Watch-PAT 200 and 24-h ABPM | 1 night + 24 h |
| Hoshide et al., 2022 | Japan | Multicenter cross-sectional study | 330 | 66.8 ± 11.9 | male 68.5% | Adults with suspected sleep-disordered breathing from 8 institutes | Suspected SDB; mean 3% ODI 21.0 ± 15.0/h | Hypertension in 65.8% | Multicenter overnight SOMNOtouch RESP monitoring | 1 night |
| Hediger-Parolini et al., 2025 | Switzerland | Prospective observational feasibility study | 10 | 55,median(IQR 45.25 – 57.5) | 4/6 | Newly diagnosed OSA patients starting CPAP with remote wearable monitoring | Diagnosed OSA; AHI > 15 events/h; baseline median AHI 29.2 [25.5-42.5] | Not specifically reported | Geneva Pulmonary League; home monitoring with Aktiia bracelet, ActiGraph Insight Watch, and DreamStation CPAP data during CPAP initiation | 42 days |
| Chen et al., 2025 | China | Real-world observational screening cohort study | 1054 analyzed (1,056,494 screened) | 43.7 ± 8.6 | 1038/16 | Nationwide smart-device consumers; 19,563 high-risk cases identified after large-scale screening | Confirmed OSA: mild 69, moderate 181, severe 730, unknown 74 | Hypertension in 19.83% of confirmed OSA | Nationwide app-based consumer screening with HUAWEI smartwatches and bracelets | >=14 days |
| Yu et al., 2024 | China | Multicenter cross-sectional study | 2573 (OSA group: 1426; Control group:1147) | 72.5, mean | 1012/1561 | Elderly hypertensive adults in primary care | OSA detected in 87.1%; moderate-to-severe OSA in 55.4% | 100% hypertensive | 15 primary care organizations in Ningxia; cloud-based analysis of data from a single-lead wearable ECG device | 72 h ECG |
| Correa et al., 2017 | Brazil | Cross-sectional observational study | 81(AHI<15 group: 55; AHI≥15 group: 26) | 42 ± 6 | 22/59 | Asymptomatic obese adults with normal casual BP and no major comorbidities | AHI < 15 (n = 55) versus >= 15 (n = 26), moderate-to-severe OSA in 32.1% | No prior hypertension; nocturnal and masked hypertension assessed by ABPM | Outpatient clinic; Watch-PAT plus Spacelabs 90207 ABPM | 1 night + 24 h |
| Kabir et al., 2024 | Canada | Prospective observational cohort study | 58 | 54.7 ± 13.5 | 33/25 | Adults referred for PSG; 16 had prior cardiovascular disease | No OSA 3, mild 12, moderate 19, severe 24; mean AHI 33.2 ± 25.7/h | Not specifically reported | In-laboratory PSG with simultaneous recording from The Patch acoustic wearable | 1 night |
| Svedmyr et al., 2016 | Germany/Sweden | Multicenter cross-sectional observational study | 440 | 55 ± 12 | male 64% | Adults referred for suspected sleep apnea or monitoring of sleep-disordered breathing | Mean AHI 19 ± 19/h; mild 30%, moderate 22%, severe 22% | Hypertension in 52% (n = 227) | Five sleep centers; finger pulse oximeter module (ChipOx) used during home or hospital-based overnight sleep studies | Overnight (>=4 h) |

Abbreviations: ABPM, ambulatory blood pressure monitoring; AHI, apnea-hypopnea index; BP, blood pressure; CPAP, continuous positive airway pressure; CV, cardiovascular; ECG, electrocardiography; ODE, oxygen desaturation event; ODI, oxygen desaturation index; OSA, obstructive sleep apnea; PG/PSG, polygraphy/polysomnography; REI, respiratory event index; SDB, sleep-disordered breathing.
