# Supplementary material for: Wearable Devices for Monitoring and Management of Comorbid Obstructive Sleep Apnea and Hypertension: Scoping Review
Source: JMIR Mhealth Uhealth. 2026 Jul 31;14:e84506. doi: 10.2196/84506 (PMC13427068; doi:10.2196/84506)
Supplement: Multimedia Appendix 3 [file mhealth-v14-e84506-s003.docx]

**Device characteristics, monitored metrics, reference standards, and key findings.**

| **Author, year** | **Wearable device** | **Sensor technology** | **Sleep/respiratory metrics** | **Cardiovascular-related metrics** | **Clinical application** | **Reference standard** | **Key finding** |
| --- | --- | --- | --- | --- | --- | --- | --- |
| Huang et al., 2021 | Morpheus Ox + CNAP | PPG-based portable sleep monitoring + beat-to-beat finger BP monitoring | TST, ODT, REI, TST90, mean SpO2, lowest SpO2 | Beat-to-beat SBP/DBP, SBPV/DBPV, hypoxia SBP index, event-related delta SBP | Quantification of ODE-related nocturnal BP fluctuations in OSA | Type-3 portable monitor (Alice PDx) for OSA confirmation | Greater ODE severity was associated with higher nocturnal SBP surges and greater BP variability. |
| Strassberger et al., 2021 | Finger pulse oximeter module (ChipOx) | Finger PPG/pulse oximetry with advanced pulse-wave analysis | SpO2-I, T < 90, TSD; alongside AHI/ODI from overnight sleep study | PWA-I, PPT, RRPO, PR-I, CRI | Overnight cardiovascular risk stratification in suspected OSA | Standard overnight PG/PSG plus ESC/ESH CV risk matrix | CRI added predictive value beyond age, sex, and BMI and outperformed AHI/ODI for identifying high cardiovascular risk. |
| Traiwannakij et al., 2026 | SOMNOtouch NIBP + WatchBP 03 | ECG + finger PPG for PTT-derived cuffless BP; cuff-based ABPM comparator | AHI, 3% ODI, SpO2 nadir, TST90 | PTT-derived nocturnal SBP/DBP; ABPM daytime/nighttime BP | Screening for nocturnal and masked hypertension in suspected OSA | Full-night PSG and 24-h ABPM | PTT-derived BP weakly correlated with ABPM, overestimated nighttime BP in moderate-to-severe OSA, and PTT-SBP ≥ 104 mmHg showed high sensitivity for nocturnal hypertension. |
| Orendain et al., 2025 | Fitbit activity tracker | Consumer wearable sleep/activity sensing with resting heart rate capture | Sleep duration, sleep variability, minutes asleep, minutes awake, sleep efficiency, social jet lag | Resting heart rate; hypertension outcome from self-report | Digital risk stratification for OSA risk and self-reported hypertension in a decentralized cohort | Berlin questionnaire for OSA risk; self-reported hypertension | Greater sleep variability, rather than average sleep duration, was associated with higher odds of both high OSA risk and self-reported hypertension. |
| Kuwabara et al., 2016 | Triggered nocturnal BP monitor (pulse oximetry + HEM-780 cuff monitor) | Pulse oximetry with oxygen-trigger algorithm plus cuff oscillometric BP | SpO2, oxygen desaturation episodes, 3% ODI, AHI | Hypoxia-peak SBP, mean nocturnal SBP, nocturnal SBP surge, DBP, pulse rate | Detection of hypoxia-induced nocturnal BP surges in OSA | Two-night PSG plus fixed-interval nocturnal BP monitoring | Hypoxia-peak SBP was much higher than mean nocturnal SBP and showed comparable reproducibility across two nights. |
| Cho et al., 2015 | Watch-PAT 200 | Peripheral arterial tonometry, pulse oximetry, pulse-rate sensing, actigraphy | AHI, ODI, lowest SaO2, total sleep time | 24-h SBP/DBP, daytime/nighttime BP, sleep-trough morning SBP surge, pre-awakening morning SBP surge | Assessment of morning BP surge in untreated hypertensive patients with OSA | 24-h ABPM; no concurrent PSG | OSA was associated with a greater sleep-trough morning SBP surge, and lower nadir oxygen saturation predicted a larger surge. |
| Hoshide et al., 2022 | SOMNOtouch RESP | ECG + finger PPG for PTT-derived cuffless BP | 3% ODI, SpO2, nasal airflow, snoring, thoracic/abdominal respiratory effort | Beat-to-beat and 30-min intermittent PTT-estimated SBP/DBP; maximum, minimum, SD, and coefficient of variation | Comparison of beat-to-beat versus intermittent nocturnal BP monitoring in suspected SDB | Office cuff BP for calibration; no external nocturnal BP gold standard | Beat-to-beat and intermittent PTT-estimated BP agreed for average BP variability indices, but beat-to-beat monitoring captured higher maximum and lower minimum BP than intermittent 30-min sampling. |
| Hediger-Parolini et al., 2025 | Aktiia bracelet + ActiGraph Insight Watch + DreamStation CPAP data | PPG wrist BP monitoring; actigraphy/accelerometry for sleep and activity | Total sleep time, sleep latency, WASO, sleep efficiency, physical activity | Daytime/nighttime SBP/DBP, heart rate, CPAP usage time | Remote longitudinal monitoring of CPAP-treated OSA under real-world conditions | Ambulatory polygraphy for OSA diagnosis; oscillometric cuff calibration for Aktiia | Remote monitoring was feasible, but nocturnal BP accuracy and data completeness were lower than daytime measurements, and manual synchronization remained burdensome. |
| Chen et al., 2025 | HUAWEI smartwatches (GT2/GT3) and bracelets (B6/4 Pro/6/7) | Wrist PPG, pulse oximetry, heart-rate sensing | Estimated OSA risk, sleep duration, SpO₂, time with SpO₂ <90% | Abnormal rhythm detection, suspected AF, ectopy, high/low heart rate | Large-scale real-world screening of OSA and arrhythmia-related risk | HSAT/PSG for OSA confirmation; clinical evaluation, 12-lead ECG, or 24-h Holter for arrhythmia confirmation | PPG-based smart devices identified high-risk OSA users at scale, and confirmed OSA cases frequently had wearable-detected abnormal rhythms or suspected arrhythmia. |
| Yu et al., 2024 | Single-lead wearable ECG device (Model 401) | Single-lead ECG with ACAT-based CVHR analysis | Estimated AHI and OSA severity across 3 nights | HRV metrics (LF/HF, SDNN) and arrhythmias such as SVES, AT, and AV block | Community-based OSA screening in elderly hypertensive patients | No concurrent PSG; ACAT-based ECG screening with physician waveform review | Wearable ECG screening identified a high OSA burden, and HRV and arrhythmia abnormalities were associated with moderate-to-severe OSA. |
| Correa et al., 2017 | Watch-PAT + Spacelabs 90207 ABPM | PAT, actigraphy, pulse oximetry, and cuff-based ambulatory BP monitoring | AHI, RDI, ODI, mean/min oxygen saturation, REM sleep | 24-h/day/night SBP/DBP, BP load, nocturnal dipping, masked hypertension | Identification of ambulatory and nocturnal BP abnormalities in asymptomatic obese adults with OSA and normal office BP | 24-h ABPM | Moderate-to-severe OSA was associated with higher 24-h, daytime, and nighttime BP and greater nocturnal DBP load despite normal casual BP. |
| Kabir et al., 2024 | The Patch | Embedded microphone + accelerometer | Tracheal respiratory sounds, snoring, SaO₂ drops, PSG-scored apneas/hypopneas/arousals | HSIS1, HSIS2, HRHS, correlations with heart rate and blood pressure | Assessment of cardiovascular burden of respiratory event-related hypoxia | In-laboratory PSG; ECG-derived heart rate | Increases in HSIS1 and HSIS2 after respiratory event termination correlated with the magnitude of hypoxemia and concurrent changes in heart rate and blood pressure. |
| Svedmyr et al., 2016 | Finger pulse oximeter module (ChipOx) | Finger photoplethysmography/pulse oximetry | AHI and sleep stages (wake, NREM, REM) | Pulse propagation time (PPT) as an overnight marker of arterial stiffness | Nocturnal vascular stiffness assessment in suspected sleep apnea | Overnight polygraphy/polysomnography; no external arterial-stiffness gold standard | Overnight PPT was shorter in hypertensive patients and independently associated with hypertension; higher AHI was also associated with shorter PPT. |

Metrics and terminology are reported as described in the original studies; reference standards and signal definitions varied across studies.

**Abbreviations:** ABPM, ambulatory blood pressure monitoring; ACAT, auto-correlated wave detection with adaptive threshold; AF, atrial fibrillation; AHI, apnea-hypopnea index; AT, atrial tachycardia; AV block, atrioventricular block; CRI, cardiac risk index; CV, cardiovascular; CVHR, cyclic variation of heart rate; DBP, diastolic blood pressure; ECG, electrocardiography; ESC/ESH, European Society of Cardiology/European Society of Hypertension; HRHS, heart rate from heart sounds; HRV, heart rate variability; HSAT, home sleep apnea test; HSIS1, first heart sound intensity; HSIS2, second heart sound intensity; LF/HF, low-frequency/high-frequency ratio; ODE, oxygen desaturation event; ODI, oxygen desaturation index; ODT, total time of oxygen desaturation events; OSA, obstructive sleep apnea; PAT, peripheral arterial tonometry; PG, polygraphy; PPG, photoplethysmography; PPT, pulse propagation time; PR-I, pulse rate acceleration index; PSG, polysomnography; PTT, pulse transit time; PWA-I, pulse wave attenuation index; RDI, respiratory disturbance index; REI, respiratory event index; REM, rapid eye movement; RRPO, respiratory-related pulse oscillations; SaO₂, arterial oxygen saturation; SBP, systolic blood pressure; SBPV, systolic blood pressure variability; SD, standard deviation; SDNN, standard deviation of NN intervals; SpO₂, peripheral oxygen saturation; SpO₂-I, hypoxia index; SVES, supraventricular extrasystole; TSD, time in symmetric desaturation; TST, total sleep time; TST90, sleep time with oxygen saturation below 90%; WASO, wake after sleep onset.
