# Supplementary material for: Wearable Devices for Monitoring and Management of Comorbid Obstructive Sleep Apnea and Hypertension: Scoping Review
Source: JMIR Mhealth Uhealth. 2026 Jul 31;14:e84506. doi: 10.2196/84506 (PMC13427068; doi:10.2196/84506)
Supplement: Multimedia Appendix 4 [file mhealth-v14-e84506-s004.docx]

**Diagnostic or Stratification Thresholds Across Included Studies**

| **Author, year** | **Device** | **OSA/sleep-respiratory thresholds** | **BP/cardiovascular thresholds** |
| --- | --- | --- | --- |
| Huang et al., 2021 | Morpheus Ox + CNAP | AHI≥5 events/h for OSA confirmation; REI severity: mild 5 to <15 events/h, moderate 15 to <30 events/h, severe ≥30 events/h; ODE defined as SpO₂ drop ≥3%. | SBP surge defined as ΔSBP≥10 mmHg. |
| Strassberger et al., 2021 | ChipOx finger pulse oximeter | No device-specific OSA cutoff reported. ODI based on ≥4% desaturations/h. | CRI 0 to 1; higher values indicate higher CV risk. |
| Traiwannakij et al., 2026 | SOMNOtouch NIBP + WatchBP 03 | OSA severity: mild 5 to <15 events/h; moderate 15 to <30 events/h; severe ≥30 events/h. | Nocturnal hypertension: nighttime BP ≥120/70 mmHg; daytime hypertension: daytime BP ≥135/85 mmHg; optimal PTT-SBP cutoff for nocturnal hypertension: ≥104 mmHg. |
| Orendain et al., 2025 | Fitbit activity tracker | No device-specific OSA cutoff reported. OSA risk categorized as low versus high by Berlin Questionnaire. | Hypertension defined by self-report. |
| Kuwabara et al., 2016 | Triggered nocturnal BP monitor | Entry screening: 3%ODI >15/h plus symptoms; AHI severity: mild 5 to <15 events/h, moderate 15 to <30 events/h, severe ≥30 events/h. | Hypoxia-induced surge nocturnal hypertension: mean nocturnal SBP ≥120 mmHg plus hypoxia-peak SBP ≥160 mmHg. |
| Cho et al., 2015 | Watch-PAT 200 | OSA defined as AHI ≥5; ODI defined as desaturation ≥3%/h. | Untreated hypertension defined by ABPM SBP >135 mmHg and/or DBP >85 mmHg. |
| Hoshide et al., 2022 | SOMNOtouch RESP | No device-specific OSA cutoff reported. SDB burden stratified by 3%ODI tertiles: 0 to 11.3/h, 11.4 to 24.9/h, and 25.1 to 74.4/h. | No explicit BP diagnostic cutoff reported. |
| Hediger-Parolini et al., 2025 | Aktiia bracelet + ActiGraph Insight Watch + DreamStation CPAP | Eligibility required AHI>15 events/h; conventional OSA severity thresholds referenced as mild 5 to <15 events/h, moderate 15 to <30 events/h, severe ≥30 events/h. | No explicit BP diagnostic cutoff reported. |
| Chen et al., 2025 | HUAWEI smartwatches and bracelets | High OSA risk: >80% of monitored nights with AHI ≥30 events/h; intermediate risk: >80% with 15<AHI<30 events/h; low risk: >80% with 5<AHI≤15 events/h. Hypoxia severity: mild SpO₂ 85 to 89%; moderate 80 to 84%; severe <80%. | High/low heart rate: resting BPM ≥100 or ≤50. Moderate-risk premature beat: ectopy 0.5% to 10% of monitored rhythm time; high risk: >10%. |
| Yu et al., 2024 | Single-lead wearable ECG device | OSA severity: normal <5 events/h; mild 5 to <15 events/h; moderate 15 to <30 events/h; severe 30 to <75 events/h; extremely severe ≥75 events/h. Final severity based on the maximum AHI across 3 nights; AHI <15 versus ≥15 used for control versus moderate-to-severe grouping. | HRV thresholds: LF/HF 1 to 3 considered normal; SDNN >100 ms considered normal. |
| Correa et al., 2017 | Watch-PAT + Spacelabs 90207 ABPM | Group 1: AHI <15 events/h; Group 2: AHI ≥15 events/h. | Masked hypertension: casual BP <140/90 mmHg with 24-h BP >130/80 mmHg, awake BP >135/85 mmHg, and/or sleep BP >120/70 mmHg; abnormal BP load: >30% of valid ABPM readings above normal limits. |
| Kabir et al., 2024 | The Patch | No OSA ≤5; mild >5 to ≤15 events/h; moderate >15 to ≤30 events/h; severe >30 events/h; apnea: >90% reduction for ≥10 s; hypopnea: >30% reduction for >10 s with arousal or ≥3% SaO₂ drop. | No explicit BP diagnostic cutoff reported. |
| Svedmyr et al., 2016 | ChipOx finger pulse oximeter | No device-specific OSA cutoff reported. | No explicit PPT or BP diagnostic cutoff reported. |

Abbreviations: ABPM, ambulatory blood pressure monitoring; AHI, apnea-hypopnea index; BPM, beats per minute; BP, blood pressure; CRI, cardiac risk index; CV, cardiovascular; DBP, diastolic blood pressure; LF/HF, low-frequency/high-frequency ratio; ODI, oxygen desaturation index; ODE, oxygen desaturation event; PPT, pulse propagation time; PTT, pulse transit time; REI, respiratory event index; SpO₂, peripheral oxygen saturation; SBP, systolic blood pressure; SDNN, standard deviation of NN intervals.
